# Using Theoretical Domains Framework (TDF) to understand implementation of a pragmatic clinical trial in Public Dental Service in Norway

**DOI:** 10.1186/s12913-021-06590-2

**Published:** 2021-07-16

**Authors:** E. A.S. Hovden, Rasa Skudutyte-Rysstad

**Affiliations:** Oral Health Center of Expertise in Eastern Norway (OHCEE), Oslo, Norway

**Keywords:** Public Dental Service, Theoretical Domains Framework, Evidence-based practice, Clinical service research

## Abstract

**Background:**

Most dental research in Norway has traditionally been conducted by universities, and the involvement of clinicians in research projects has not been a common practice.

The aim of the present study was to identify behavioral factors that influence effective implementation of a pragmatic clinical trial in the Public Dental Service (PDS) in Norway and to understand which of these factors result in higher patient recruitment.

**Methods:**

Dentists, dental hygienists, and dental assistants at nine Public Dental Service clinics in three counties in Norway involved in an ongoing pragmatic clinical trial were asked to complete an electronically distributed questionnaire based on the Theoretical Domains Framework (TDF).

**Results:**

Thirty-seven out of 69 dentists and dental hygienists (54 %) and seventeen out of 57 dental assistants (30 %) answered the questionnaire. “Knowledge” was the domain with the highest mean response, suggesting strong confidence in personal knowledge and practical skills among the clinicians. Together with “beliefs about consequences,” “organizational resources,” and “environmental context,” “knowledge” was the one of five domains identified as important behavioral determinants in patient recruitment to clinical trials by dental professionals.

**Conclusions:**

The findings suggest that TDF was useful to understand factors affecting implementation of clinical trials in PDS and that several factors such as clinical relevance of trial to be implemented, organizational resources, and communication with the research team require more attention when planning and implementing clinical trials in PDS.

## Background

For just a decade ago most of dental research in Norway has traditionally been conducted by universities and the involvement of clinicians in research projects has not been a common practice [1, 2]. Increased focus on evidence-based practice during the last decades has revealed substantial knowledge gaps and a need for clinical research in most fields of dentistry both in Norway and internationally [1, 3, 4]. Thus, to increase research activities among clinicians and use of clinical data for research, practice-based research networks in dentistry have been established in several countries [5, 6], and an increasing number of studies involving clinicians in primary dental care have been published during the last decade [7, 8]. In order to increase research activity and quality assurre the clinical practice in Public Dental Service (PDS) in Norway, five Oral Health Centers of Expertise (OHCE) were established by the the Norwegian government in 2007 [9]. The OHCEs are partly owned by the PDS in their respective regions and are (among other tasks) assigned to initiate and conduct clinical practice-based research in the PDS [9]. Furthermore, in 2017 the Norwegian health authorities presented “Research strategy in dentistry” [1], emphasizing again the need for increased research activities in primary dental service and closer collaboration between academic researchers and clinicians [1].

The main tasks of PDS in Norway are [1] to provide free dental care for children, adolescents, dependent elderly, and other groups of patients with special healthcare needs, and [2] to carry out community-based preventive activities and oral health promotion [10]. Although PDS provides regular dental care to many patients, there is little tradition of dental clinicians using patient data in quality assurance and research [11]. There are only a few studies in Norway in which clinicians in PDS have contributed to data collection [12–14]. Available questionnaire surveys on Norwegian dental clinicians’ interests in participating in and contributing to research projects [15, 16] revealed that clinicians agree that their participation in research would be beneficial to patient treatment and say they would be interested in being involved in research. However, as contributing to research is not a common task for dental clinicians [10], conducting research projects at PDS clinics requires changes in accepted practices which can be challenging and time consuming. Changes in both individual and collective behavior in PDS are required [17] for effective implementation of research activities; thus, it is important to understand what factors can influence these behavioral changes [18].

Theoretical Domains Framework (TDF) is a method of comprehensively identifying perceived psychological and organizational factors that may influence the implementation of evidence-based behavior of healthcare professionals [19, 20]. The framework has been previously applied in dentistry to explore factors influencing preventive behaviors of general dental practitioners [21–23] and has been validated as a suitable method for theoretical assessment of behavioral change in implementation research [24]. In this study, the TDF framework is for the first time used to identify factors influencing implementation of a pragmatic clinical trial in PDS in Norway.

The aim of the present study was to identify behavioral factors influencing effective implementation of a pragmatic clinical trial in the PDS in Norway and to assess the utility of TDF in understanding which of these factors result in higher patient recruitment.

## Materials and methods

### Participants/respondents

Respondents in this study were clinicians involved in an ongoing pragmatic clinical trial concerning the effectiveness of resin-based fissure sealant (RBFS) and fluoride varnish (FV) in preventing occlusal caries in first permanent molars of high-risk children. The two methods are routinely applied for caries management in high-risk children; however, the choice of method is to a large extent dependent on clinicians’ preferences.

Dentists, dental hygienists, and dental assistants at nine PDS clinics in three counties were involved in the trial, designed and initiated by a research team from the Oral Health Center of Expertise in Eastern Norway (OHCEE). Clinicians identified high-risk children during routine examinations. After a written informed parental consent, children received baseline intervention in which FV and RBFS were randomly applied on contra-lateral teeth using split-mouth design, and each child served as his/her own control.

Prior to the trial, the research team held introductory meetings with clinicians at all nine clinics focusing on study protocol and patients recruitment. Each clinic had a locally appointed coordinator responsible for the trial, and every clinician could contact the research team when needed.

To evaluate the process of implementing pragmatic clinical trials in PDS clinics, all involved clinicians (69 dentists and dental hygienists and 57 dental assistants) were asked to complete a questionnaire based on the TDF framework.

### TDF-based questionnaire

The TDF framework developed by Cane et al. [24] and Huijg et al. [25, 26] was used as a guide to assess behavioral factors affecting implementation of pragmatic clinical trial at PDS clinics. We based the development of the questionnaire on previous research by Skoien et al. [27] as this study had similarities to the implementation of research in the dental clinics and use the TDF *post-implementation.* As in Skoien et al. [27] we excluded the domain “emotion” from the questionnaire as we didn’t find the question from Huijg et al. [26] relevant to our study. Furthermore, as limited time resources have been previously shown to be a barrier for implementing research into practice [28–30] we divided the domain “environmental context and resources” into “organisational resources” and “environmental context” [25]. In the end ten of TDS domains were included in the questionnaire. These were: “knowledge”, “skills”, “professional role and identity”, “beliefs about capabilities”, “motivation”, “beliefs about consequences”, “intentions”, “organizational resources”, “environmental context”, and “social influences”. The items within each domain were based on previous research on factors influencing implementation of interventions in health care services [26, 27]. The items were adapted to be relevant for implementation of this particular clinical trial in PDS in Norway. The questions regarding knowledge and skills in using FV and RBFS treatment methods were applied only to clinicians involved in patient treatment: dentists and dental hygienists.

The responses to the questionnaire were scored on a five-point Likert scale with the alternatives “strongly agree,” “agree,” “neither agree or disagree,” “disagree,” and “strongly disagree.” The alternatives were assigned numerical attitude scores from 1 (strongly disagree) to 5 (strongly agree). A higher attitude score indicated a more positive attitude. For the negatively framed questions (32 and 42), the numerical scores were reversed.

Scores for items comprising each domain were summed up and an average domain score was calculated for each respondent. An estimate of internal consistency (Cronbach’s alpha) was calculated for each domain.

The background characteristics of clinicians comprised profession (dentist, dental hygienist, or dental assistant), years of clinical experience, first time involvement in research projects (yes/no), size of clinic, full or part-time position, and number of recruited participants. Years of clinical experience were dichotomised into 9 years or less and 10 years or more. Clinic size was defined based on the number of dentists and dental hygienists, with small clinics having up to 6 clinicians and large ones having 7 or more clinicians.

The questionnaire was distributed via local project coordinators who forwarded an internet link to the Questback software to all employees at the respective clinics. For non-responders, two reminders were sent. Data collection was performed during December 2019–January 2020, just after completing patient recruitment to the trial .

Data were processed and analyzed using SPSS statistical program package. Frequency distributions, means, standard deviations (SD), and range were used for descriptive statistics. Student’s t-test was used to compare differences in the mean domain scores in relation to independent variables. The level of statistical significance was set at 5 %.

**Fig. 1:**
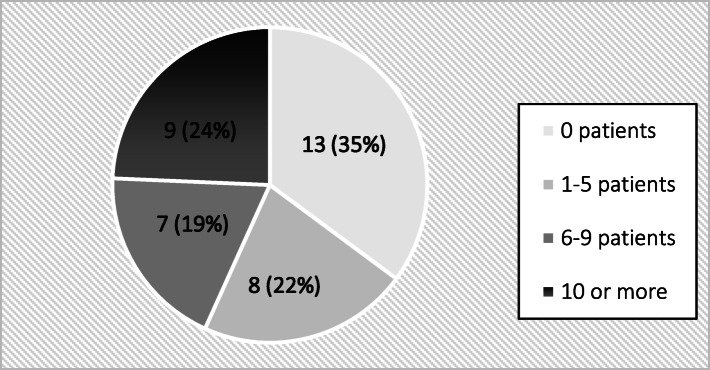
Distribution of dentists and dental hygienists (n = 37) according to the number of patients recruited to the clinical trial

As, one third of the clinicians (35%) (Fig. 1) have not recruited any patients, the Zero-inflated Poisson (ZIP) regression models in STATA statistical program package were used to assess the factors influencing patient recruitment. To show that the ZIP model fitted the data better than the standard Poisson regression model (PRM), we plotted the observed counts versus what the two models predicted. This showed that the PRM over-predicted zero counts. In addition, a formal test for comparing the ZIP with the PRM using the Vuong test showed that the ZIP was better (*P* = 0.0028) to use for our data. For further analysis, the unadjusted ZIP models were first fitted to the data to determine the explanatory variables that were used in the adjusted models. Second, three separate adjusted ZIP models based on variables with *P* < 0.20, *P* < 0.10, and *P* < 0.05 were fitted to the data. Akaike information criterion (AIC) was used to choose the best adjusted model from among the three. The AIC states that among a selection of nested models, the model with the smallest AIC value should be used. In our case, this was the model based on explanatory variables with *P* < 0.10 in the univariate analyses.

The study was evaluated by the data protection officer at Viken County and the Norwegian Centre for Research Data (reference 727,424). All responses in the study were anonymized.

## Results

Thirty-seven of 69 dentists and dental hygienists (54 %) and seventeen of 57 dental assistants (30 %) responded to the TDF-based questionnaire. Background characteristics of participants (Table 1) show that the majority of clinicians had over 10 years of clinical experience, worked at large clinics in full-time positions, and had no previous experience of participating in research projects.

**Table 1:** Background characteristics of clinicians

	**Dentists and dental hygienists (***n***=37)**	**Dental assistants(***n***=17)**
**Clinical experience**		
** ≥10 years**	23 (62%)	11 (65%)
** <10 years**	14 (38%)	6 (35%)
**First time involvement in a research project**		
** Yes**	21 (57%)	14 (82%)
** No**	16 (43%)	3 (18%)
**Clinic size**		
** Large**	24 (65%)	10 (59%)
** Small**	13 (35%)	7 (41%)
**Position**		
** Full time**	27 (73%)	14 (82%)
** Part time **	10 (27%)	3 (18%)

Of 37 dentists and dental hygienists who responded to the TDF questionnaire, 24 (65 %) have recruited patients to the clinical trial. The mean number of recruited patients was 7 (SD = 11) with a range from 0 to 48 recruited patients (Fig. 1).

### Responses to the TDF domains

All dentists and dental hygienists (100 %) had knowledge about use of RBFS and FV (Table 2). 97 % had knowledge about advantages of using RBFS and 95 % had knowledge about advantages of using FV. In comparison, 87 % had knowledge about disadvantages of RBFS whereas 68 % responded that they were aware of disadvantages of FV. Most of dentist and dental hygienists (92% and 86 %) were confident in using RBFS and FV in management of occlusal caries (Table 2).

**Table 2:** TDF domains, project implementation questions, and frequency distributions of participants (dentists and dental hygienists (*n* = 37) and dental assistants (*n *= 17)) responding “agree” and “strongly agree” to the questionnaire items. RBFS: resin-based fissure sealants; FV: fluoride varnish

Domain	Question	Dentists and dental hygienists	Dental assistants
		**% agree/strongly agree**	**% agree/strongly agree**
**Knowledge**	1. I know how to apply RBFS. 2. I know how to apply FV. 3. I am aware of advantages of RBFS. 4. I am aware of disadvantages of RBFS. 5. I am aware of advantages of FV. 6. I am aware of disadvantages of FV.	100 % 100 % 97 % 87 % 95 % 68 %	n/a n/a n/a n/a n/a n/a
**Skills**	7. I am confident in using RBFS for prevention of occlusal caries. 8. I am confident in using FV for prevention of occlusal caries. 9. I am aware of what contributing to a research project means. 10. It was simple to work with the split-mouth method.	92 % 86 % 70 % 44 %	n/a n/a 88 % n/a
**Professional role and identity**	11. Contributing to research projects is part of my work duties.	65 %	53 %
**Beliefs about capabilities**	12. I was confident about my role in the clinical trial. 13. I was clear about expectations of me in the clinical trial. 14. I felt safe to ask patients to participate in the clinical trial. 15. I had overview of patients that could be recruited to the clinical trial. 16. It was easy to do proper follow-up of participants in the clinical trial.	84 % 68 % 84 % 68 % 35 %	35 % 29 % 53 % 59 % 47 %
**Motivation**	17. I was highly motivated to contribute to the clinical trial before project start. 18. The whole team was highly motivated before the clinical trial started. 19. I was highly motivated to contribute to the clinical trial through the project period. 20. The whole team was highly motivated to implement the clinical trial through the project period.	41 % 47 % 41 % 19 %	53 % 59 % 53 % 41 %
**Beliefs about consequences**	21. The clinical trial topic was highly relevant for patients. 22. The clinical trial topic was highly relevant for me. 23. I expect the results from the clinical trial will be useful to my clinical practice. 24. It was rewarding for me as clinician to contribute to a research project.	84 % 89 % 84 % 57 %	71 % 59 % 71 % 41 %
**Intentions**	25. I intend to participate in future research projects. 26. I intend to motivate my colleagues to participate in future research projects.	70 % 70 %	53 % 59 %
**Organizational resources**	27. I had enough time for implementing the project. 28. *I often felt that I needed to prioritize other tasks above the implementation of the research project*^a^*.* 29. I had enough time to adapt the project to my daily practice.	43 % 24 % 35 %	35 % 41 % 24 %
**Environmental context**	30. I received enough information about the project from OHCE before project start. 31. I had good communication with OHCE during the project. 32. It was easy to recruit participants to the project. 33. Patients were motivated to participate in the project.	73 % 49 % 32 % 46 %	53 % 47 % 59 % 53 %
**Social influences**	34. The leader of my clinic was supportive of the project. 35. We discussed the project at my clinics and encouraged each other to participate. 36. My colleagues were supportive towards my work with the project. 37. *I experienced resistance from my colleagues towards my work with the project*^a^*.*	62 % 30 % 32 % 73 %	41 % 47 % 41 % 65 %

^a^reversed question, *n/a* not applicable

When asked about participation in research, the majority of respondents were aware of what it means to participate in a research project, and more than half perceived contribution to research projects to be part of their work duties.

Questions covered by the domain “beliefs about capabilities” revealed that the majority of dentists and dental hygienists had high self-confidence and expectations about recruiting patients to the trial.

The individual motivations of clinicians to contribute to the clinical trial were stable throughout the entire period of the trial. However, the motivation of the whole dental team to conduct the trial diminished through the clinical trial period.

A majority of clinicians agreed that the project topic was highly relevant for their clinical practice; however, fewer perceived that it was rewarding for them to contribute to the trial. Fewer than half agreed with the statement, “I had enough time for implementing the project.”

Questions within the domain of “environmental context” revealed that the majority of clinicians felt receiving enough information about the clinical trial from the OHCEE research team before project start. Fewer respondents felt that good communication was maintained throughout the clinical trial period.

The items covering the “social influences” domain showed that the leaders of the clinics were supportive of the trial. In spite of that, only a few clinicians experienced that their colleagues were supportive towards their work with the trial.

Analysis of the internal consistency across domains showed that seven of ten scales for dentists and dental hygienists had very good reliability (above 0.8 for two scales and above 0.7 for five scales) (Table 3). For dental assistants, Cronbach’s alpha ranged from 0.71 to 0.99, indicating high internal reliability of the measures (Table 3).

For dentists and dental hygienists, two domains, “knowledge” and “skills” had the highest mean response scores (equal to the most positive responses) with low variability. For dental assistants, “skills” was the domain with the highest mean response score and the lowest response variability. “Organizational resources” and “motivation,” were the domains with the lowest mean response scores for dentist and dental hygienists. For dental assistants the domains with the lowest mean response were “organizational resources” and “professional role and identity”.

**Table 3:** Descriptive statistics for the ten domains of the TDF scale, responses from dentists and dental hygienists (*n*=37) and dental assistants (*n*=17)

**Domain (number of questions)**	**Dentists, dental hygienistsMean (range), SD**	**Scale reliability (α)**	**Dental assistantsMean (range), SD**	**Scale reliability**
**Knowledge (6)**	4.38 (3-5), 0.55	0.890	n/a	n/a
**Skills (4)**^a^	4.03 (2-5), 0.61	0.662	4.18 (3-5), 0.64	n/a
**Professional role and identity (1)**	4.00 (3-5), 0.56	n/a	3.29 (1-5), 1.21	n/a
**Beliefs about capabilities (5)**	3.76 (2-5), 0.66	0.759	3.49 (2-4), 0.68	0.707
**Motivation (4)**	3.10 (1-4), 0.66	0.719	3.40 (3-5), 0.83	0.855
**Beliefs about consequences (5)**	3.96 (2-5), 0.67	0.824	3.85 (3-5), 0.73	0.906
**Intentions (2)**	3.84 (3-5), 0.67	0.692	3.50 (1-5), 1.12	0.988
**Organizational resources (3)**	3.05 (1-4), 0.79	0.770	3.20 (2-4), 0.71	0.881
**Environmental context (4)**	3.45 (1-5), 0.53	0.530	3.59 (2-5), 0.81	0.855
**Social influences (4)**	3.46 (3-5), 0.63	0.760	3.54 (3-5), 0.70	0.780

^a^ one question for dental assistants, *n/a* not applicable

### Factors associated with number of patients recruited in the pragmatic clinical trial

Estimates of incidence rate ratios (IRRs) obtained from the ZIP regression model are presented in Table 4. The analysis showed that dentists and dental hygienists previously involved in a research project were 1.75 times more likely to recruit patients into the project than clinicians who were involved in a research project for the first time. The number of patients recruited was 2.5 times and 3 times higher for each additional score in “beliefs about consequences” and “environmental context,” respectively. We also observed that for each additional score in “organizational resources” and “knowledge,” clinicians were more likely to recruit patients.

**Table 4:** Factors associated with number of patients recruited in the project by dentists and dental hygienists (n = 37). Estimates of incidence rate ratios (IRRs) with 95 % CI obtained from the zero-inflated Poisson regression model

Factors	Unadjusted		Adjusted	
	**IRR (95 % CI)**	***P*-value**	**IRR (95 % CI)**	***P*-value**
**Clinical experience (ref: ≤9 years)**				
**≥ 10 years**	0.96 (0.75, 1.23)	0.74		
**First time involvement in research projects (ref: Yes)**				
**No**	**1.23 (0.97, 1.57)**	**0.09**	**1.75 (1.29, 2.38)**	**< 0.01**
**Clinic size (ref: Small)**				
**Large**	0.88 (0.69, 1.11)	0.27		
**Position (ref: Part time)**				
**Full time**	1.20 (0.92, 1.56)	0.18		
**Knowledge**	1.21 (0.98, 1.48)	0.07	**1.47 (1.03, 2.08)**	**0.04**
**Skills**	**1.86 (1.52, 2.27)**	**< 0.01**	1.20 (0.68, 2.08)	0.53
**Professional role and identity**	**1.59 (1.31, 1.92)**	**< 0.01**	1.23 (0.78, 1.94)	0.37
**Beliefs about capabilities**	**1.82 (1.51, 2.20)**	**< 0.01**	1.08 (0.78, 1.51)	0.64
**Motivation**	**1.57 (1.24, 1.98)**	**< 0.01**	**1.56 (0.99, 2.44)**	0.06
**Beliefs about consequences**	**2.74 (2.16, 3.48)**	**< 0.01**	**2.46 (1.56, 3.88)**	**< 0.01**
**Intentions**	**1.39 (1.13, 1.72)**	**< 0.01**	0.74 (0.50, 1.09)	0.13
**Organizational resources**	**1.46 (1.24, 1.73)**	**< 0.01**	**1.53 (1.20, 1.95)**	**< 0.01**
**Environmental context**	**2.98 (2.46, 3.61)**	**< 0.01**	**3.00 (2.10, 4.29)**	**< 0.01**
**Social influence**	1.04 (0.87, 1.25)	0.65		

## Discussion

Pragmatic clinical trials are increasingly used in medicine and dentistry, representing “real-world” trials [29, 30]. However, little is currently known about the factors that influence clinicians’ contribution to research. Using Theoretical Domains Framework (TDF) in the present study, we were able to identify important behavioral determinants in implementation of a pragmatic clinical trial and in patients recruitment by dental professionals in the PDS. These were; “knowledge”, “ “beliefs about consequences”, “organizational resources” and “environmental context”.

Respondents in the study were dentists, dental hygienists, and dental assistants involved in an ongoing pragmatic clinical trial concerning the effectiveness of resin-based fissure sealant (RBFS) and fluoride varnish (FV) in preventing occlusal caries in first permanent molars of high-risk children. As both treatment methods are routinely used by dental professionals in their daily clinical practice, it was not surprising that the domains with the highest positive response scores were “knowledge” and “skills”. This indicates strong confidence in personal knowledge and skills in using the two treatment methods. Knowledge and skills have previously been shown to be important factors in changing the behavior of healthcare professionals [24], and they can be enablers for effective implementation of clinical trials and, in the case of the present study, of patient recruitment.

 Most dentists and dental hygienists responded that they felt safe in asking patients to participate in the trial, though fewer than one-third reported that it was easy to actually recruit patients. In practice, the recruitment of children took more time than was estimated prior to project start, and findings showed large individual variations in the number of participants recruited per clinician (range from 0 to 48).

In the present study, the “environmental context” was the domain with highest association with patient recruitment. The responses revealed that although 73 % of dentists and dental hygienists reported that they received enough information from the research team prior to the trial, fewer than half were satisfied with the communication and support received during the clinical trial. One possible explanations for these findings could be that each clinic had an appointed project coordinator who was responsible for disseminating information from the research team and acting as a link between researchers and clinicians. Although clinicians could contact the research team for questions and inquiries by phone and email, the research team was not able to contact the involved clinicians directly.

The responses suggest that direct communication was not optimal throughout the trial and that communication between clinicians and research team needs to be improved/emphasized in future studies. Lack of communication between clinicians and the OHCEE research group could be a potential barrier in patient recruitment as support and regular communication between the research team and clinicians has been shown in previous studies to be an important facilitator in patient recruitment [29].

Responses within the “beliefs about consequences” domain related to relevance and benefits of research were highly positive, with a majority of clinicians agreeing that the topic was highly relevant and that results from the clinical trial will be useful in their clinical practice. The “beliefs about consequences” domain was also strongly associated with patient recruitment in the clinical trial. This is in line with findings reported in previous studies [29–31] in which the relevance of the research project for patient care has been shown to be a facilitator of clinicians’ involvement in research.

The remaining domains strongly related to patient recruitment were “organisational resources” and “motivation,” which had the lowest response scores for all clinicians in the present study. Previous research has suggested that lack of time, unwillingness to take on additional work, and integration of the clinical trial within routine workloads at PDS may be important barriers to success [28–30]. Similarly, most dentists and dental hygienists in the present study felt that they did not have enough time for implementing the clinical trial in their daily practice. In addition to lack of time, there was a lack of team motivation throughout the clinical trial. Lack of motivation could without a doubt be a possible barrier to contributing to the trial as motivation can be reflective or automatic; it characterizes the brain processes that drive behavior and are the reason for people’s actions, willingness, and goals [17, 24, 32]. The pragmatic clinical trial was a relatively new experience for involved clinicians and was in competition with many other demanding tasks in PDS. Thus, emphasis on available time and resources and on motivation of the entire team should be key elements in planning new trials in PDS.

The questionnaire for dental assistants was modified by excluding items related to treatment methods, and their responses were analyzed separately. Although dental assistants were highly motivated and reported more positive attitudes in several domains than did dentists and dental hygienists, the domain with the lowest response scores was “professional role and identity.” A possible explanation for this could be that dental assistants were not assigned a specific role in the trial as they do not treat patients; their contribution was related to identifying high-risk children, recruitment, and follow-up. This lack of predetermined duties could explain the fact that only 35 % of dental assistants felt confident about their role, and only 29 % knew what was expected from them in the project. On the other hand, high motivation scores indicate that dental assistants have the potential to be important contributors in future projects, especially related to patient recruitment and follow-up.

The present study used an electronically distributed questionnaire for data collection, and the response rate was relatively low, especially among dental assistants. As the participation in the survey was voluntary, selection bias related to personal interests of clinicians may have occurred. It may be speculated that respondents with more positive attitudes towards the pragmatic clinical trial were more likely to respond to the survey. Moreover, including the assessment of respondents’ knowledge around the patient recruitment process and other aspects of trial participation, would probably give a better understanding of trial implementation and should be considered in the future studies. As most of the previous studies, based on TDF approach, are qualitative studies more effort should be put in developing the quantitative TDF approach. To the best of our knowledge, the present study is the first in Norway to investigate behavioural factors influencing dental clinician’s contribution to research projects by using the TDF framework.

## Conclusions

Using Theoretical Domains Framework (TDF) in the present study, we were able to identify a number of aspects within organizational and individual domains that can influence PDS professionals’ contribution to clinical trials. The research should work towards clinical trials having relevance to practice and researchers should keep in mind that individual motivation, clarity of involved clinicians’ roles and tasks, and guidance and communication with the research team are vital factors driving successful implementation of clinical trials at dental clinics.

Furthermore, TDF proved valuable in analyzing issues faced by dental teams involved in pragmatic clinical trials and in understanding clinical behaviors in the evolving area of evidence-based practice. To the best of our knowledge, the present study is the first in Norway to investigate behavioral factors influencing dental clinicians’ contribution to research projects using the TDF framework.

## Data Availability

The datasets used and/or analysed during the current study are available from the corresponding author on reasonable request.

## Abbreviations

FV: Fluoride varnish
IRRs: Incidence rate ratios
OHCE: Oral Health Centers of Expertise
OHCEE: Oral Health Center of Expertise in Eastern Norway
PDS: Public dental service
RBFS: Resin-based fissure sealant
TDF: Theoretical Domains Framework
